# Supplementary material for: Polyamine detergents tailored for native mass spectrometry studies of membrane proteins
Source: Nat Commun. 2023 Sep 14;14:5676. doi: 10.1038/s41467-023-41429-w (PMC10502129; doi:10.1038/s41467-023-41429-w)
Supplement: Supplementary file 1 — Supplementary Information [file 41467_2023_41429_MOESM1_ESM.pdf]

Supplementary Information

Polyamine detergents tailored for native mass spectrometry studies of membrane proteins

Yun Zhu,[1,†] Bo-Ji Peng,[1,†] Smriti Kumar,[1,†] Lauren Stover,[1,†] Jing-Yuan Chang,[1] Jixing Lyu,[1] Tianqi Zhang,[1] Samantha Schrecke,[1] Djavdat Azizov,[1] David Russell,[1] Lei Fang,[1,*] Arthur Laganowsky[1,*]

[1] Department of Chemistry, Texas A&M University, College Station, TX 77843
[†] These authors contributed equally to this work
* Corresponding Author: Fang@chem.tamu.edu; ALaganowsky@chem.tamu.edu

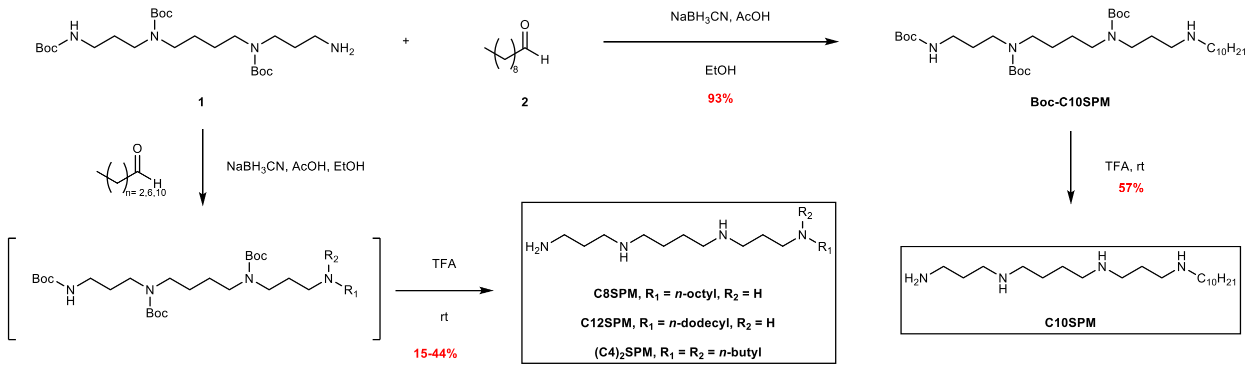

Supplementary Figure 1. Syntheses of SPM-derived detergents. The synthesis and purification of C10SPM was detailed in the main text. C8SPM, C12SPM and $(C4)_2$SPM were synthesized using a similar method without purification after the first reductive amination step to simplify the procedure.

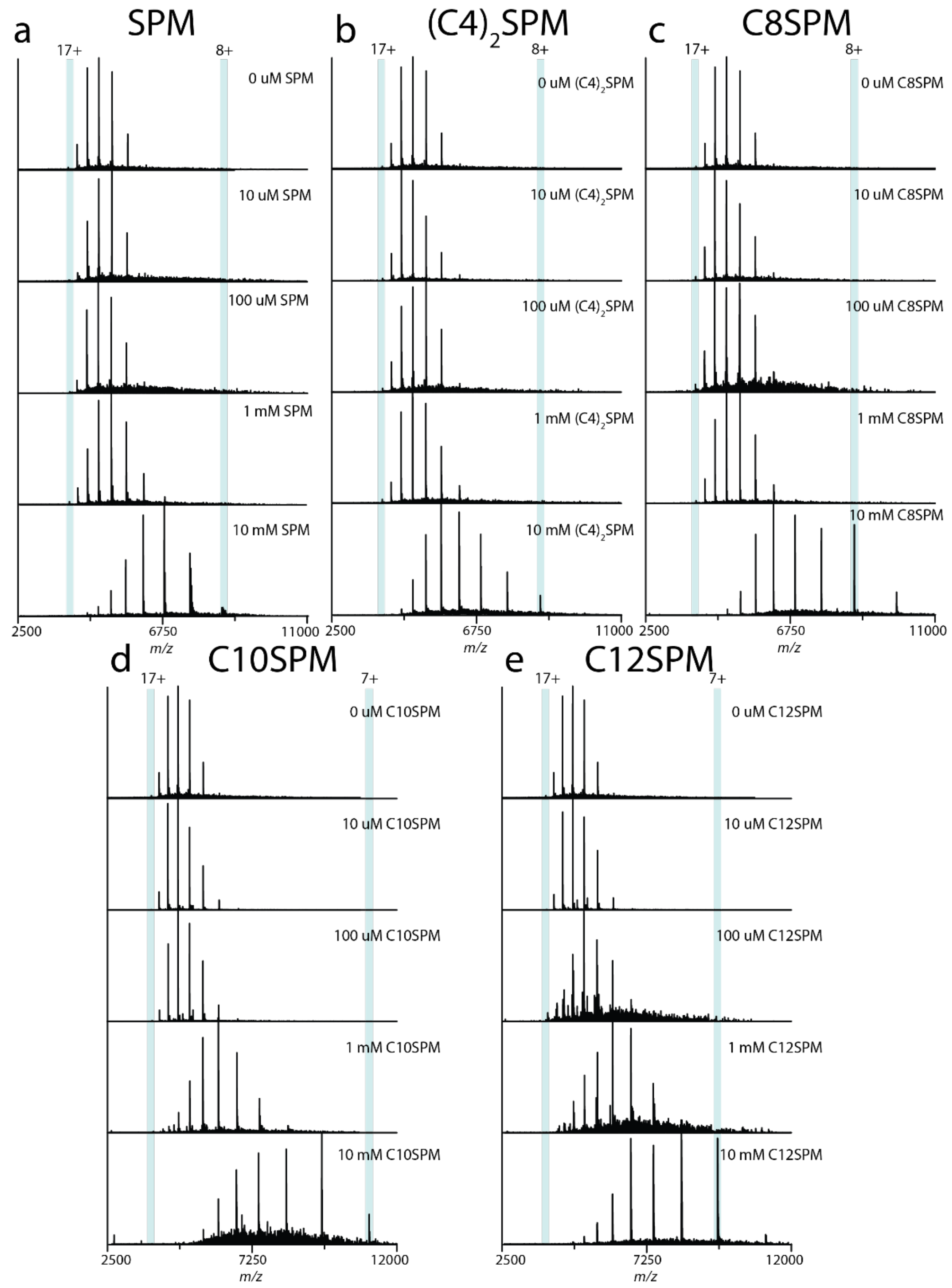

Supplementary Figure 2. Charge reduction of TRAAK with SPM and SPM detergents. a Representative mass spectrum of TRAAK in C10E5 supplemented with **a** SPM, **b** $(C4)_2$SPM, **c** C8SPM, **d** C10SPM and **e** C12SPM. The concentration of the additive is provided in the inset.

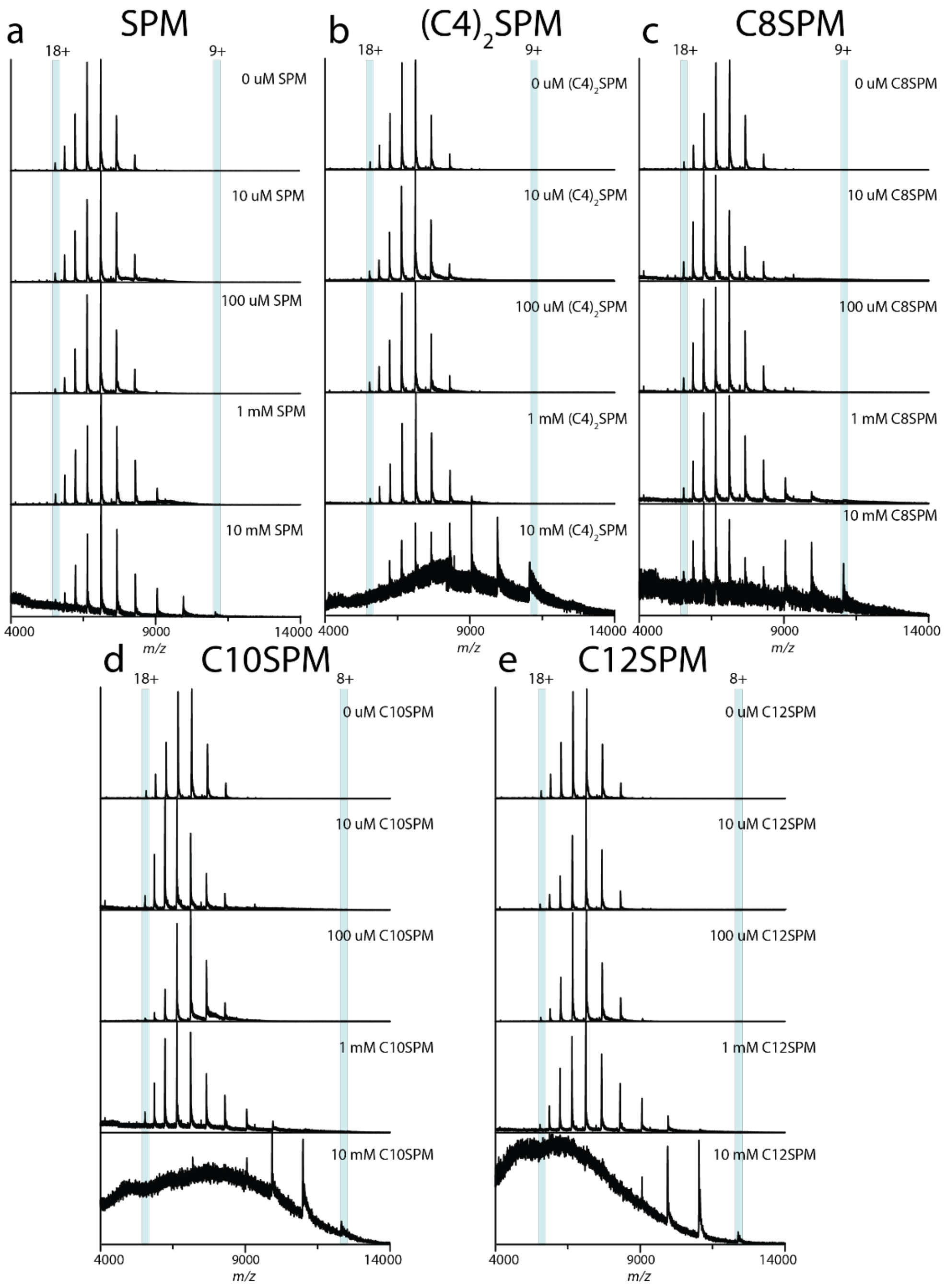

Supplementary Figure 3. Charge reduction of AqpZ with SPM and SPM-derived detergents. a Representative mass spectrum of AqpZ in C8E4 and supplemented with SPM. The concentration of SPM is provided in the inset. Mass spectrum of AqpZ in C8E4 and doped with different concentrations of **b** $(C4)_2$SPM, **c** C8SPM, **d** C10SPM, and **e** C12SPM. Shown as described for panel **a**.

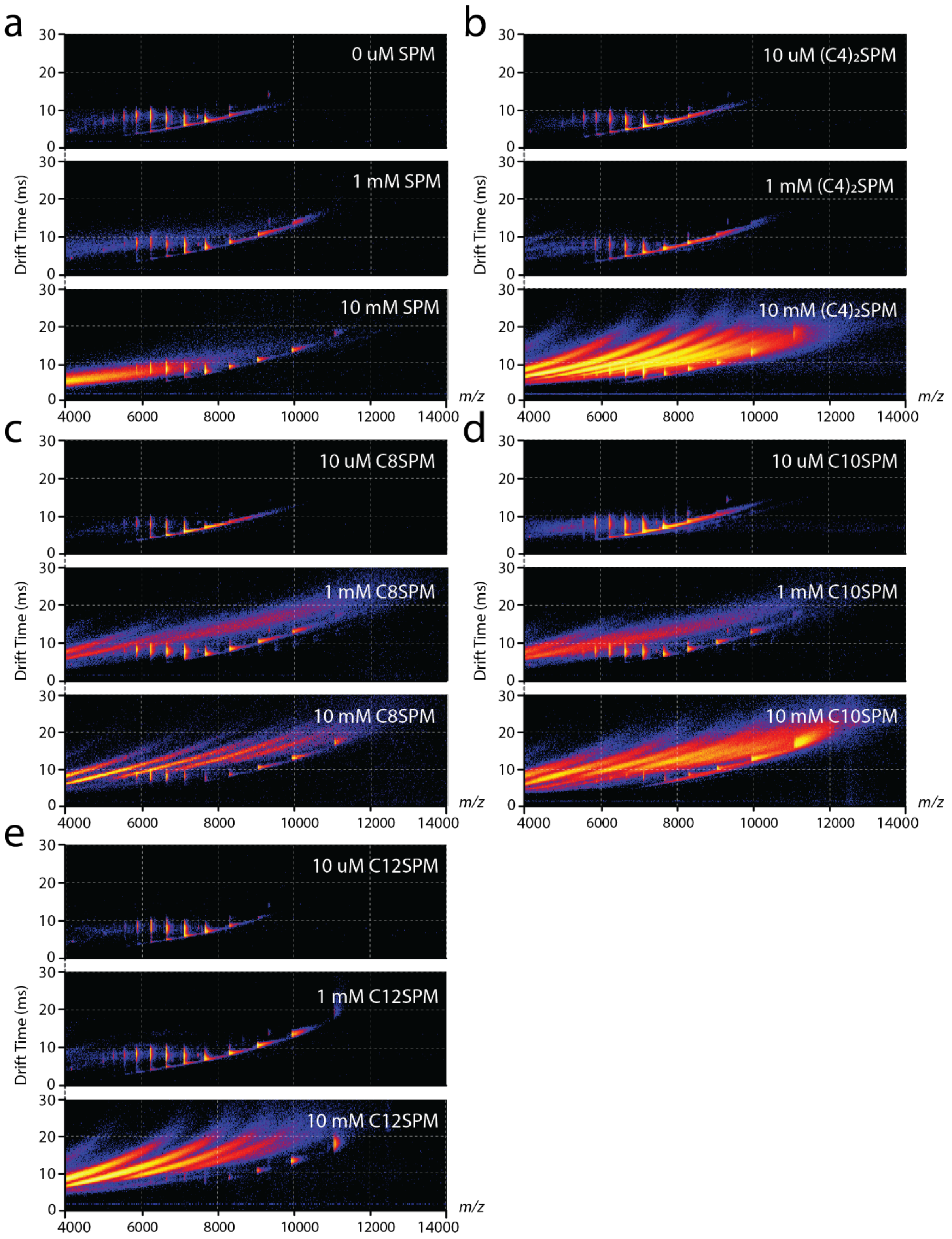

Supplementary Figure 4. Ion mobility mass spectra of AqpZ in different environments. The additive and concentration are denoted in the inset. AqpZ in C8E4 doped with varies concentration of **a** SPM, **b** $(C4)_2$SPM, **c** C8SPM, **d** C10SPM and **e** C12SPM.

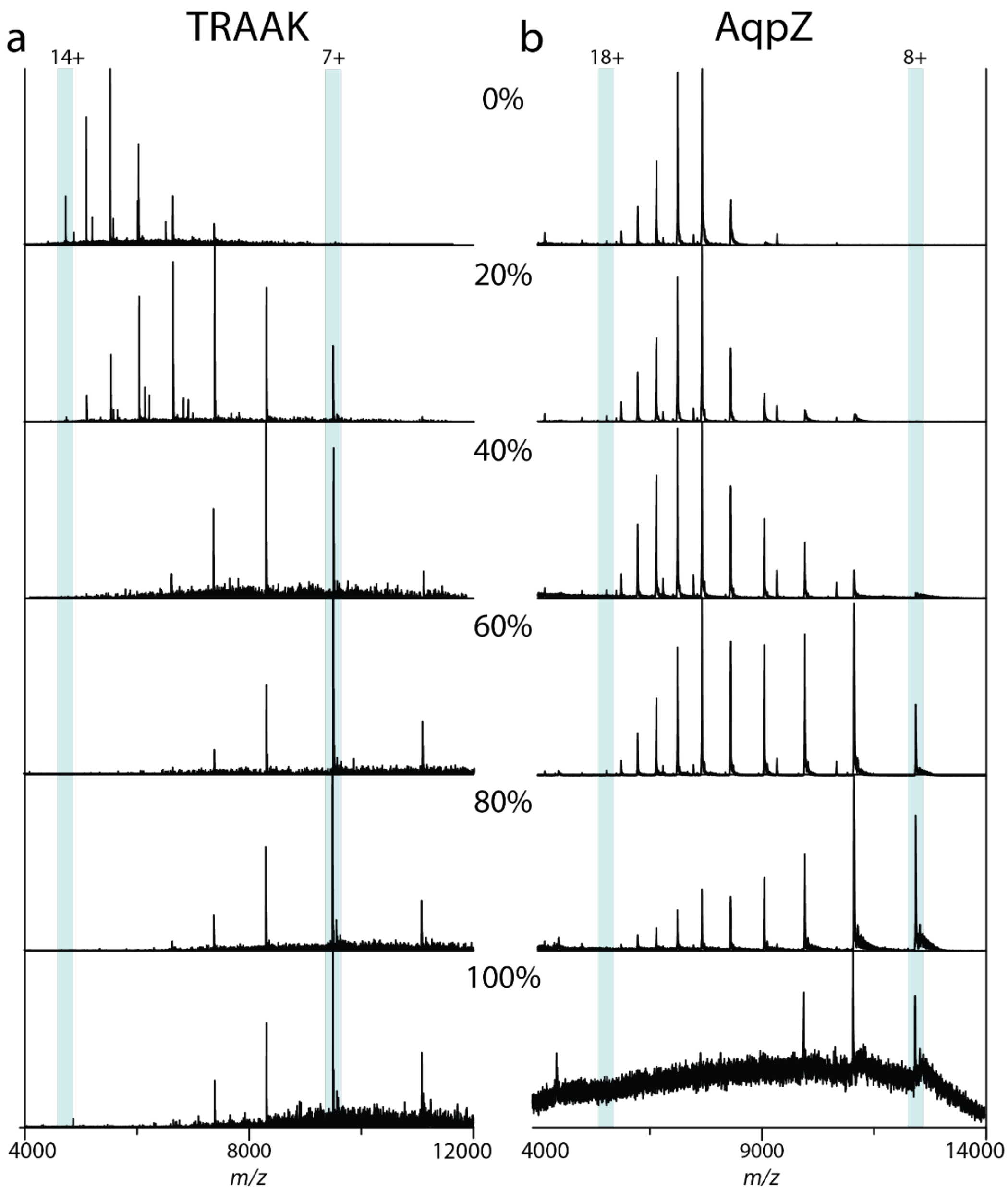

Supplementary Figure 5. Charge reduction of membrane proteins in mixed micelle environments. a-b Representative mass spectra of **a** TRAAK and **b** AqpZ in different ratios of C10SPM with C10E5 and C8E4, respectively. The percentage indicates the amount of C10SPM.

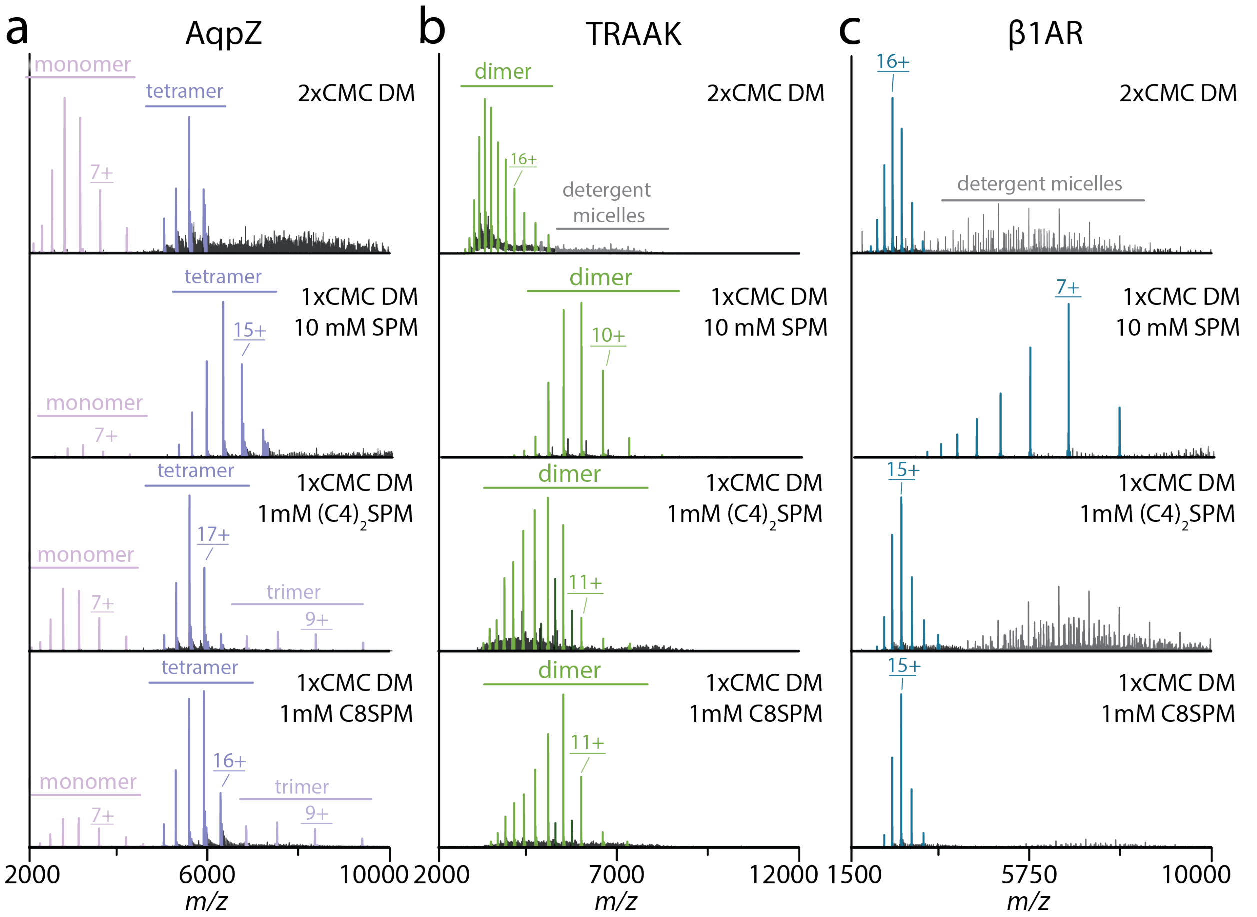

Supplementary Figure 6. Charge-reducing effect on different proteins in DM with the addition of SPM and various SPM-derived detergents. **a** AqpZ in 2 x CMC DM, 1xCMC DM with 10 mM SPM, 1xCMC DM with 1 mM $(C4)_2$SPM, 1xCMC DM with 1 mM C8SPM (from top to bottom). **b** Mass spectrum of TRAAK under different conditions. **c** Mass spectrum of β1AR under different conditions. Shown as described for **a**.

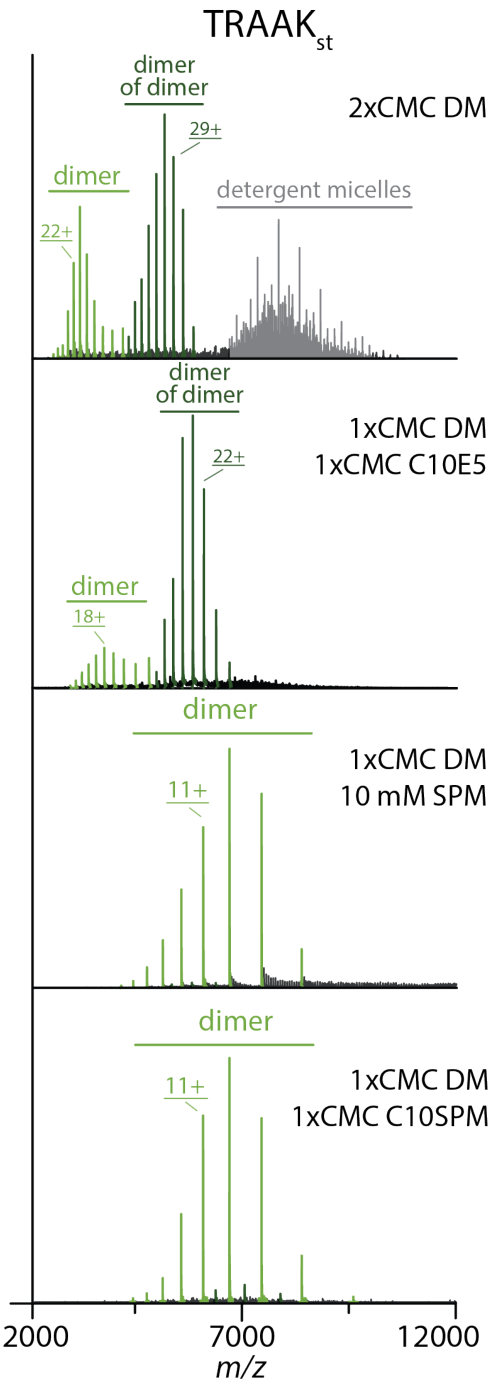

Supplementary Figure 7. TRAAK$_{st}$ solubilized in DM with the addition of SPM and C10SPM. Dimer of the homodimer was observed for TRAAK$_{st}$ in DM. Other mass spectra are for TRAAK in DM but mixed with different concentration of additives, which are denoted in the inset.

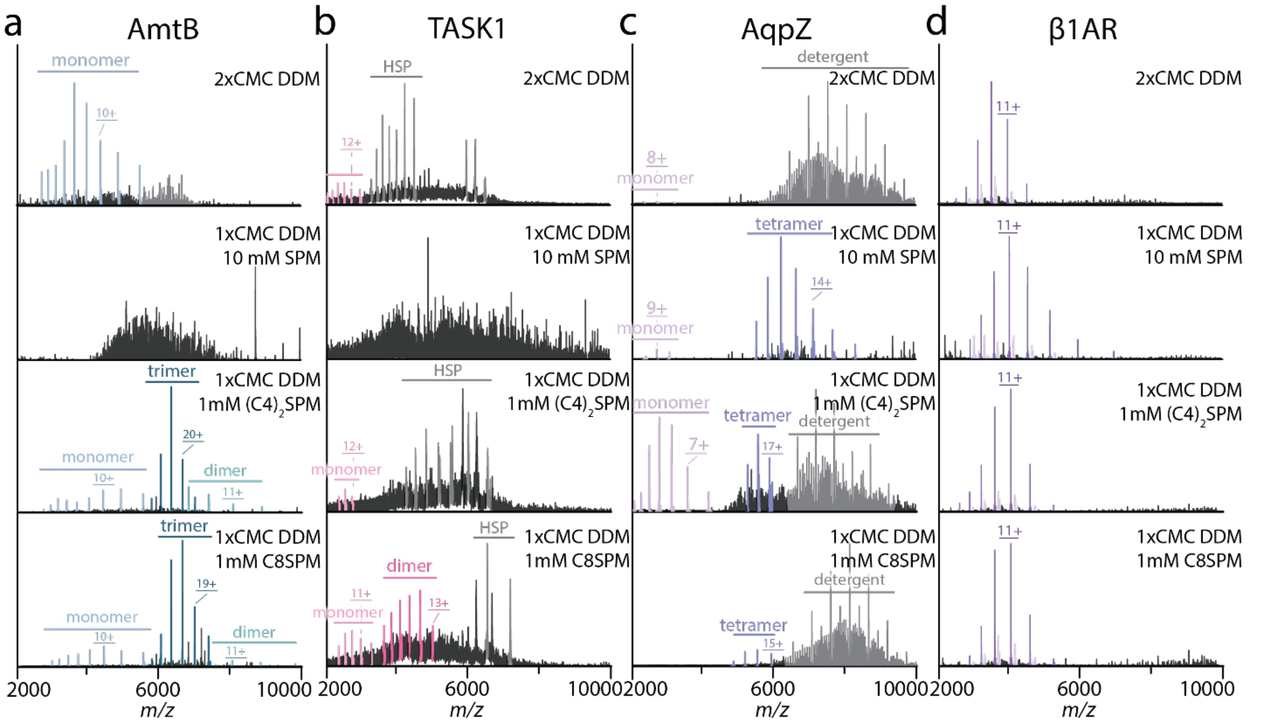

Supplementary Figure 8. Different membrane proteins solubilized in DDM and in the presence of SPM and SPM detergents. **a** AmtB in 2xCMC DDM, 1xCMC DDM with 10 mM SPM, 1xCMC DDM with 1 mM $(C4)_2$SPM, 1xCMC DDM with 1 mM C8SPM (from top to bottom). **b** Mass spectrum of TASK1 in DDM with the addition of SPM and various SPM-derived detergents. **c** Mass spectrum of AqpZ in DDM and in the presence of SPM and SPM-derived detergents. **d** Mass spectrum of β1AR in DDM and with SPM and in the presence of SPM and SPM-derived detergents. Shown as described for **a**.

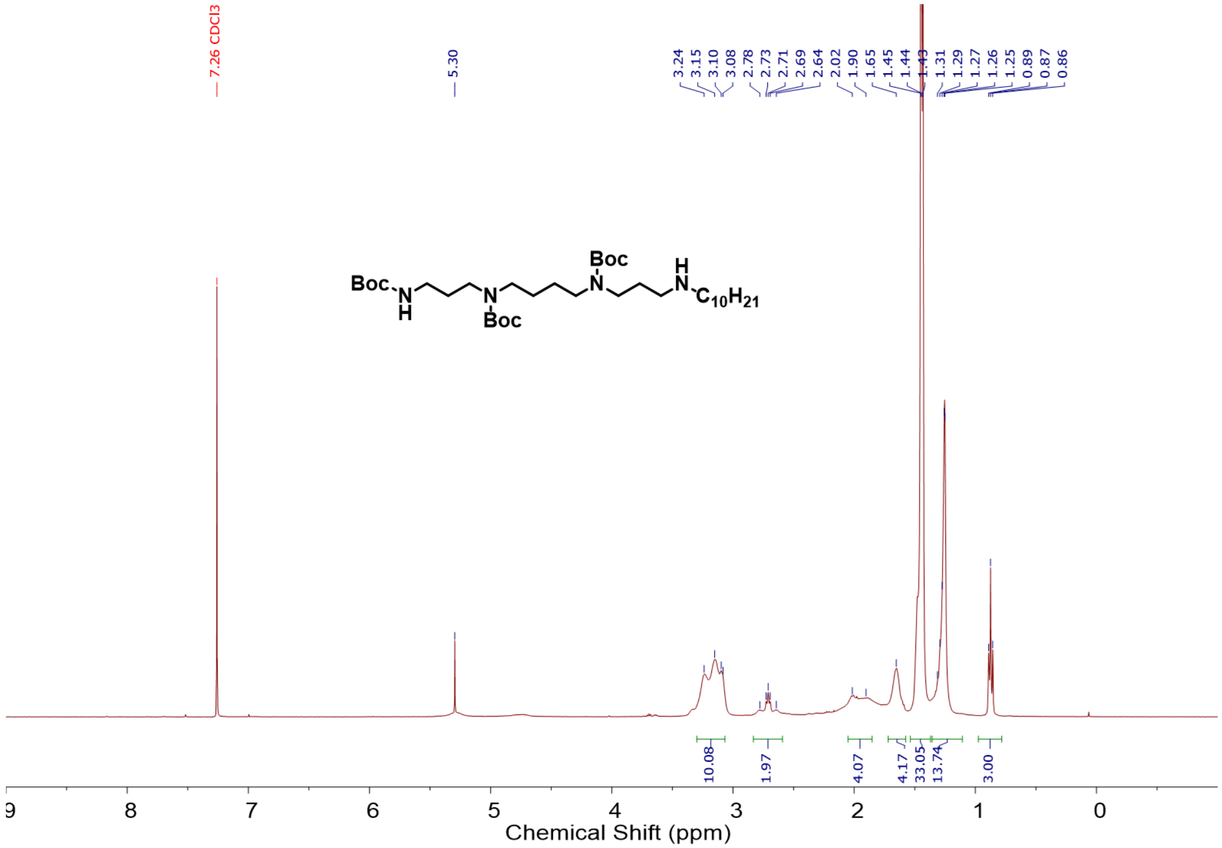

Supplementary Figure 9. ^{1}H NMR of Boc-C10SPM (400 MHz, $CDCl_3$, 298 K).

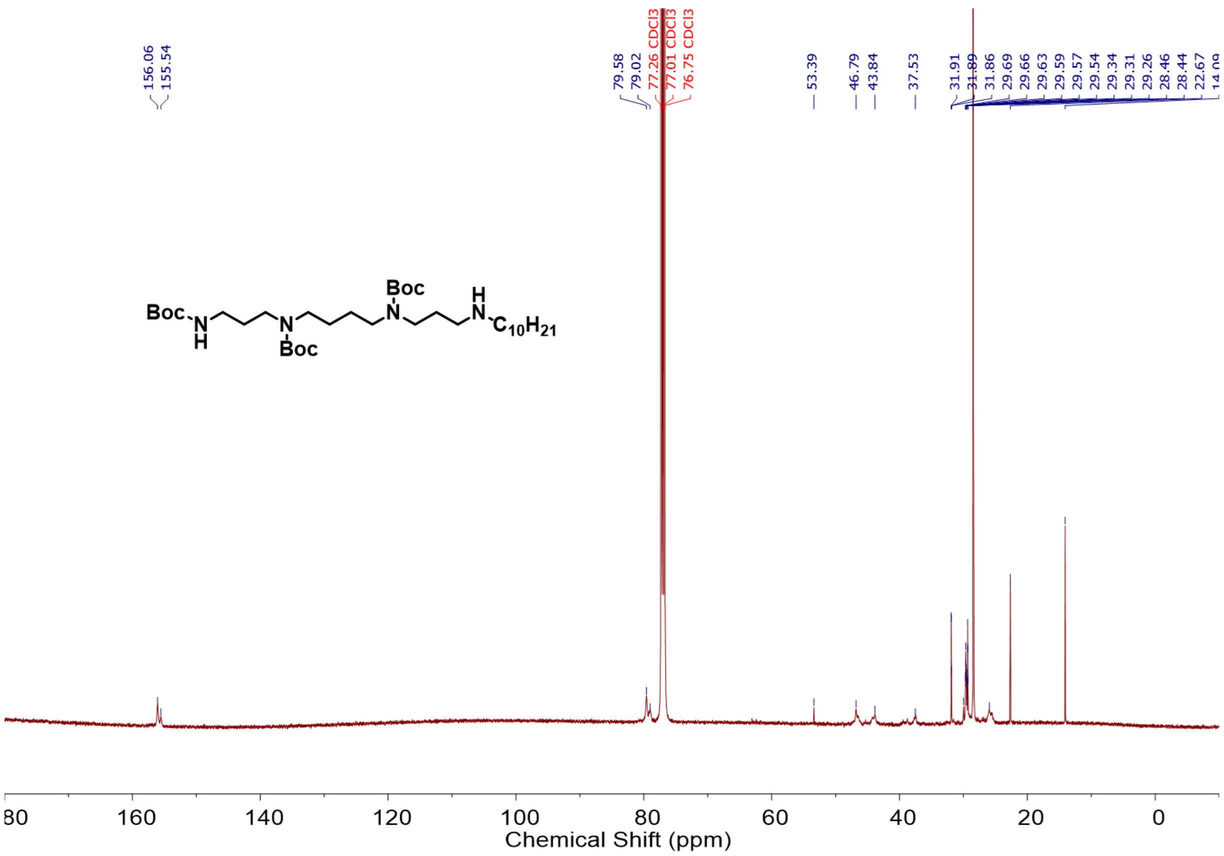

Supplementary Figure 10. ^{13}C NMR of Boc-C10SPM (126 MHz, $CDCl_3$, 298 K).

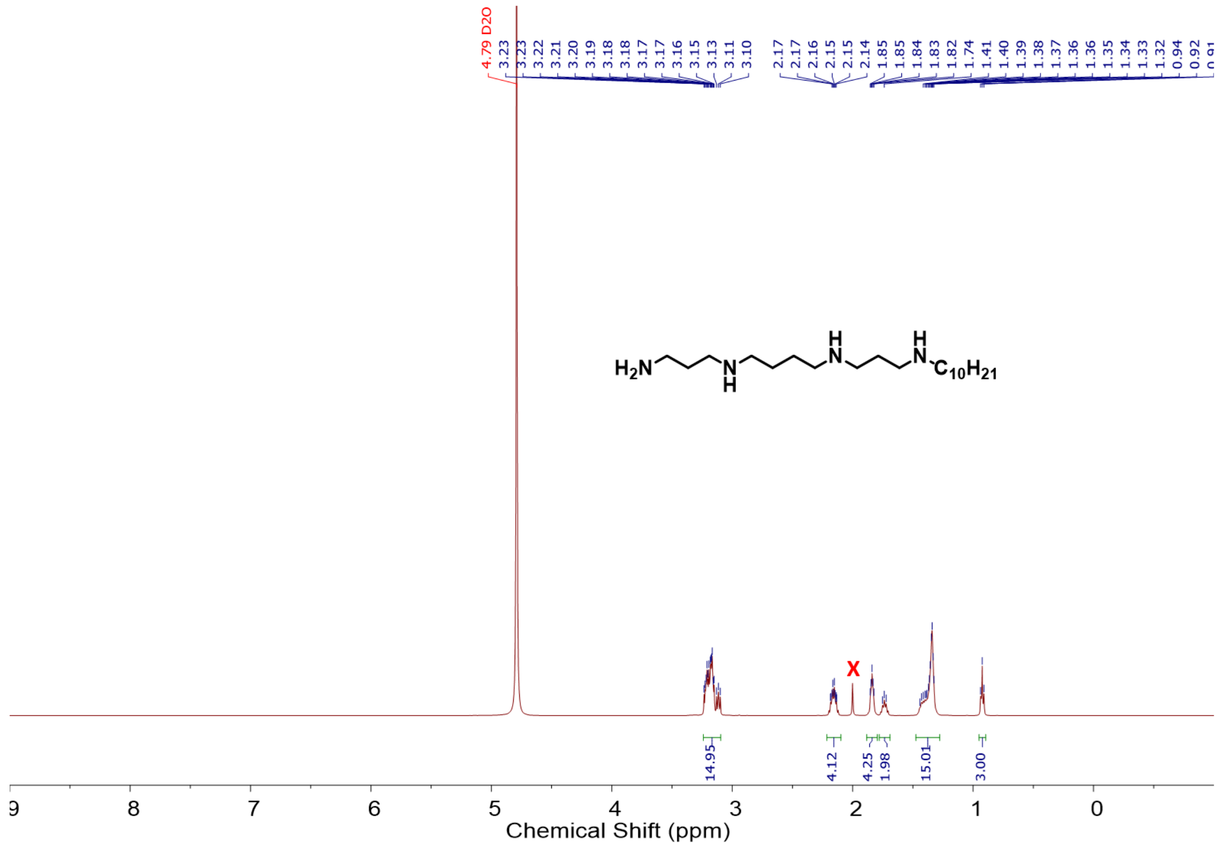

Supplementary Figure 11. ^{1}H NMR of C10SPM (500 MHz, D_2O, 298 K).

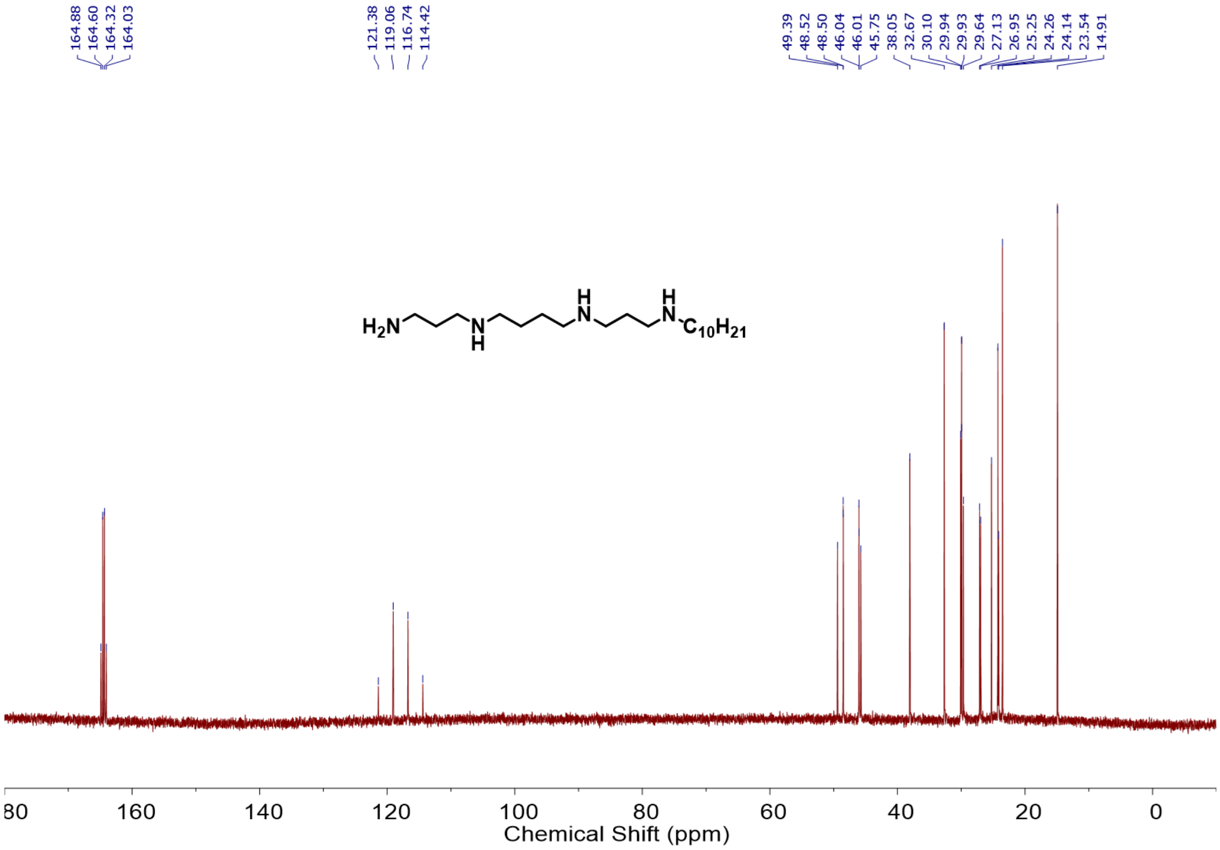

Supplementary Figure 12. ^{13}C NMR of C10SPM (126 MHz, D_2O, 298 K).

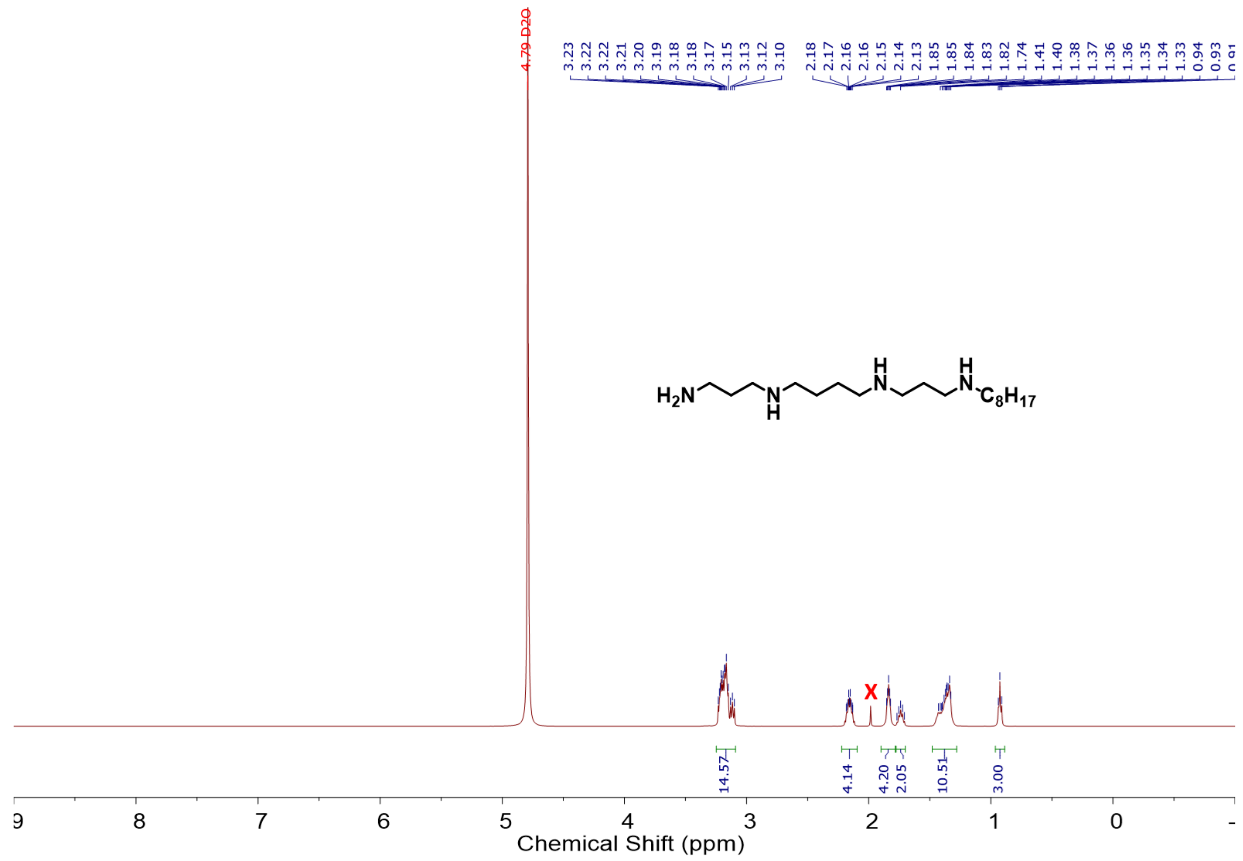

Supplementary Figure 13. ^1H NMR of C8SPM (500 MHz, D_2O, 298 K).

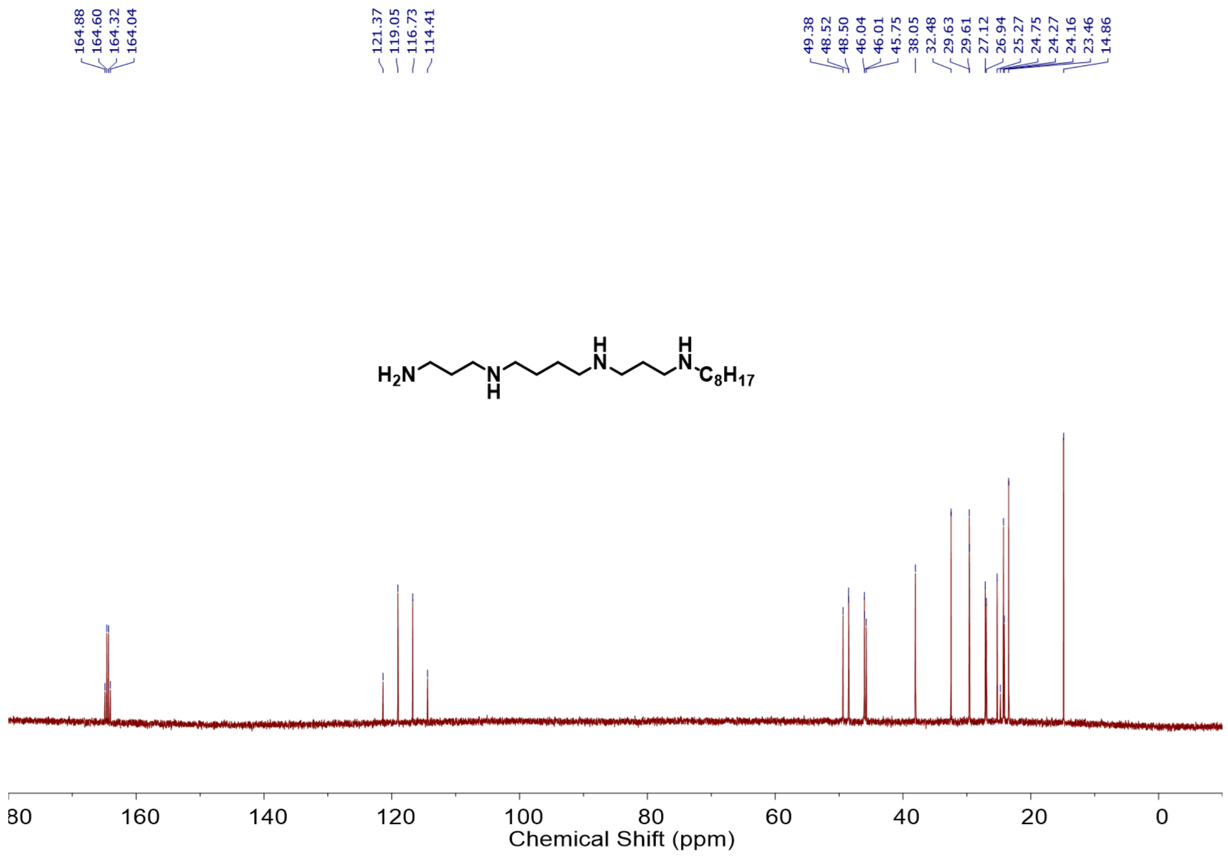

Supplementary Figure 14. ^{13}C NMR of C8SPM (126 MHz, D_2O, 298 K).

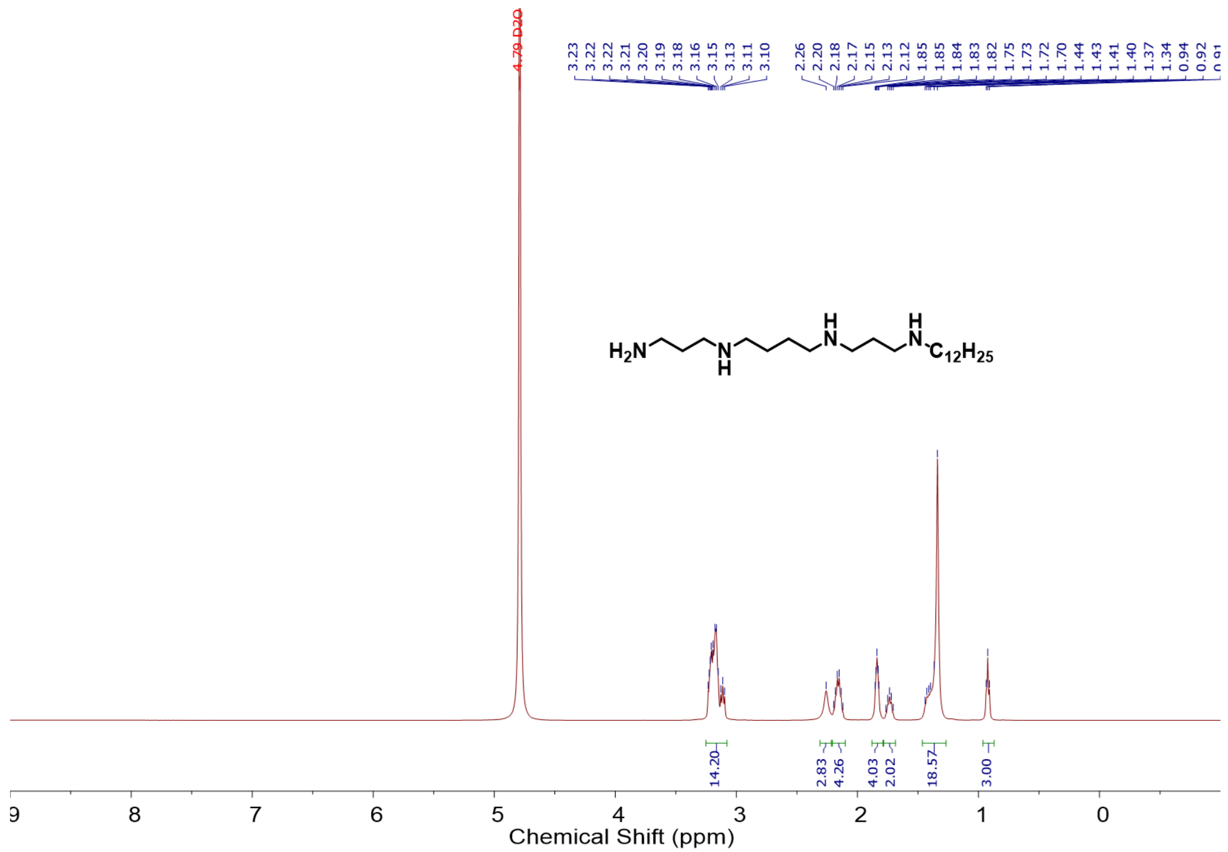

Supplementary Figure 15. ^{1}H NMR of C12SPM (500 MHz, D_2O, 298 K).

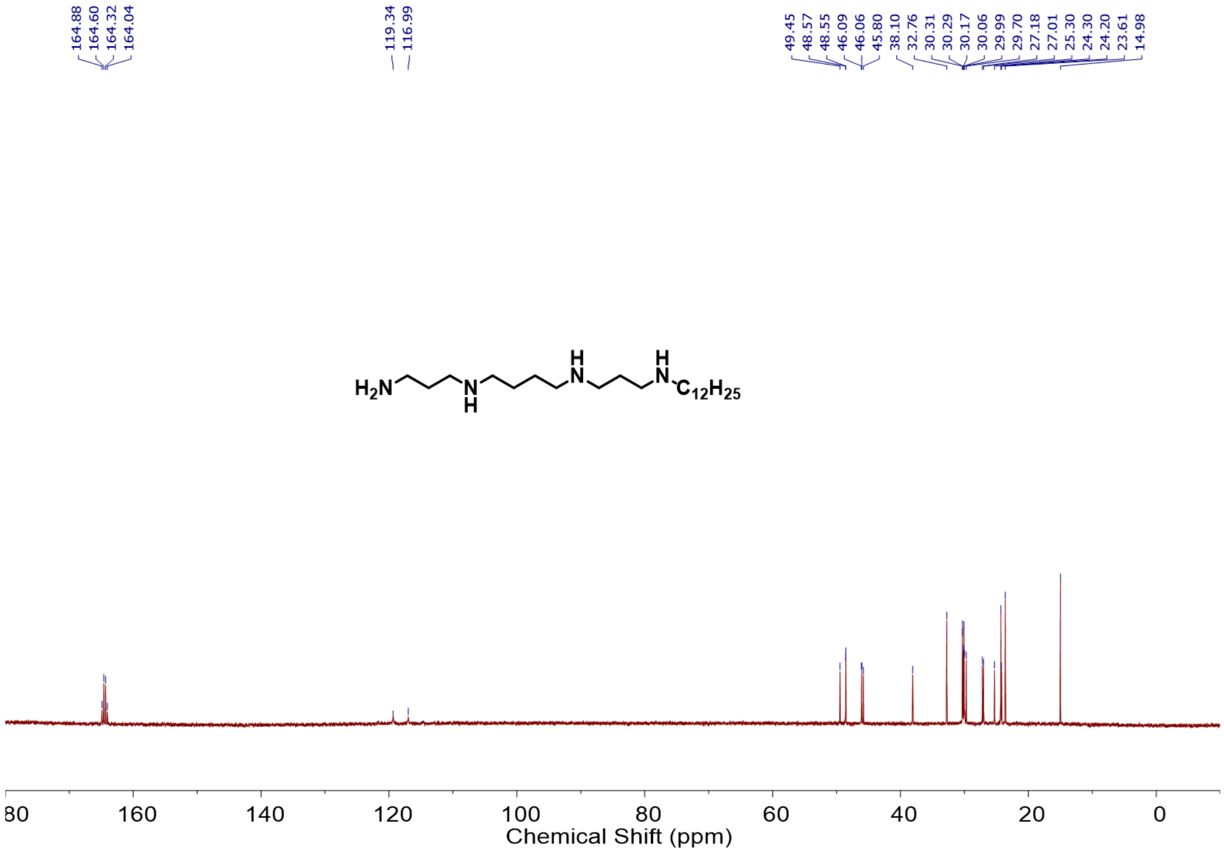

Supplementary Figure 16. ^{13}C NMR of C12SPM (126 MHz, D_2O, 298 K).

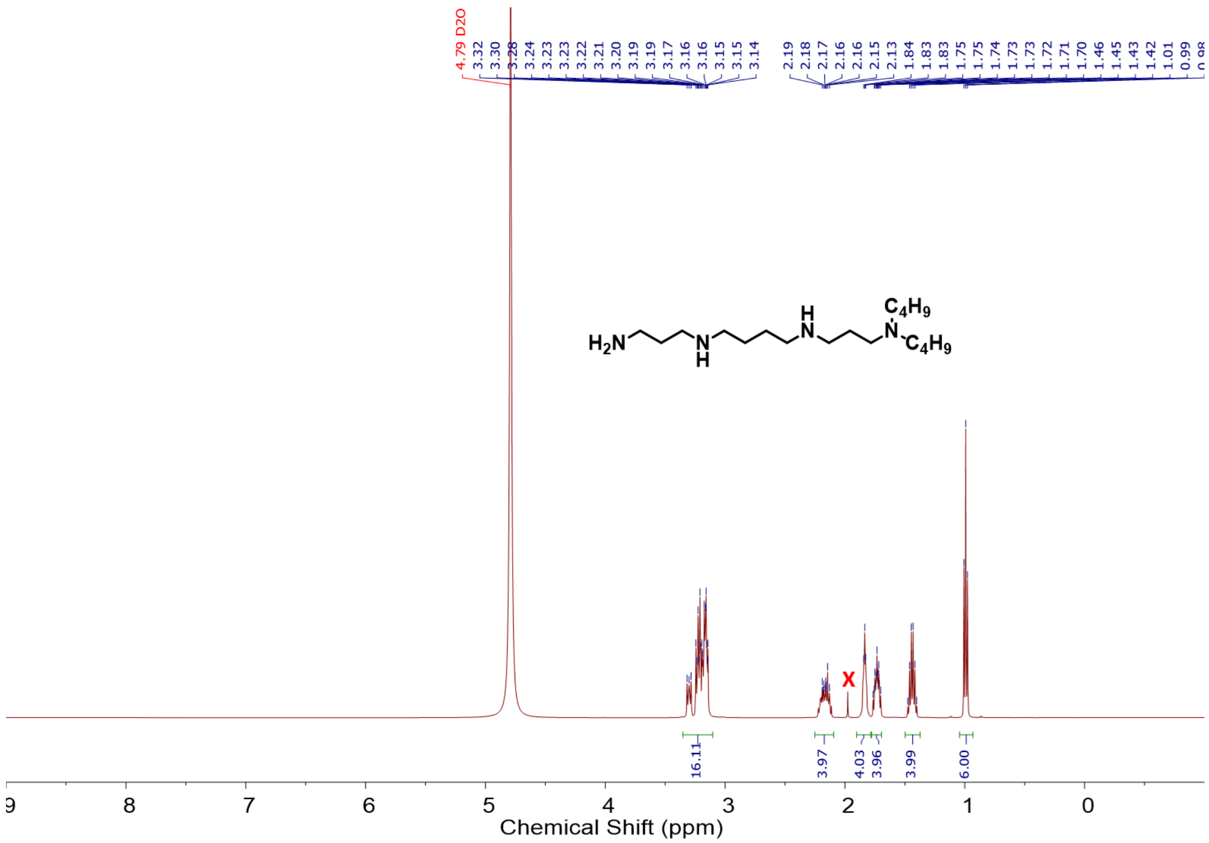

Supplementary Figure 17. ^{1}H NMR of (C4)$_2$SPM (500 MHz, D_2O, 298 K).

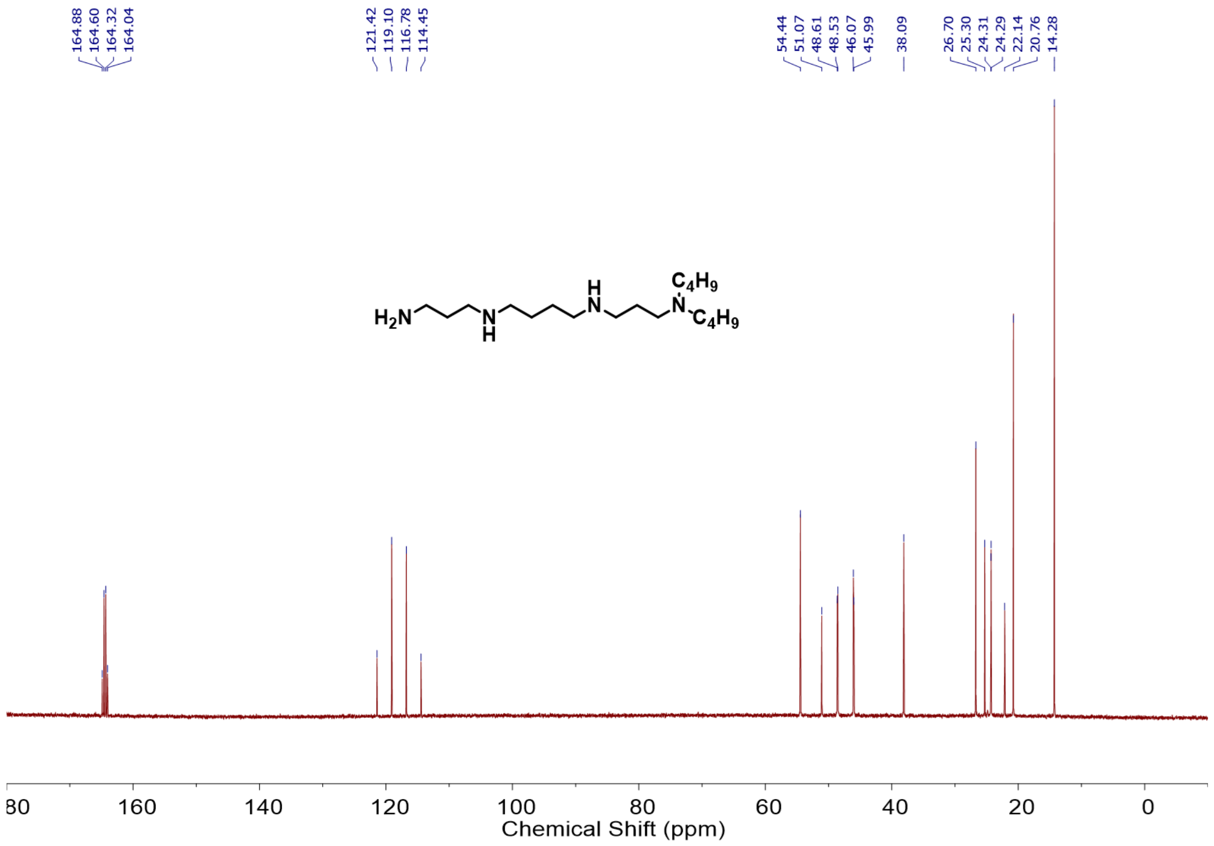

Supplementary Figure 18. ^{13}C NMR of $(C4)_2SPM$ (126 MHz, D_2O, 298 K).

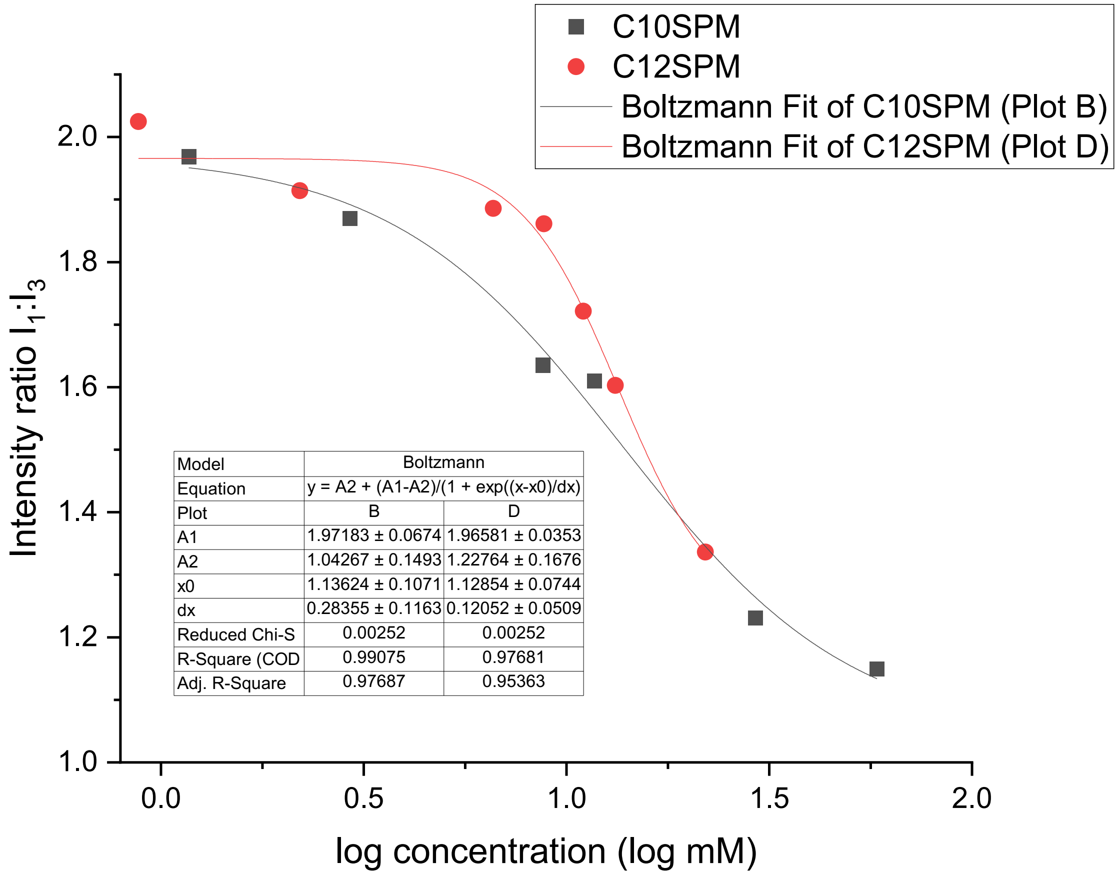

Supplementary Figure 19. I_1:I_3 ratio of pyrene as function of logarithmic concentration of C10SPM and C12SPM for CMC values determination. Both C10SPM and C12SPM are measured in a single trial.

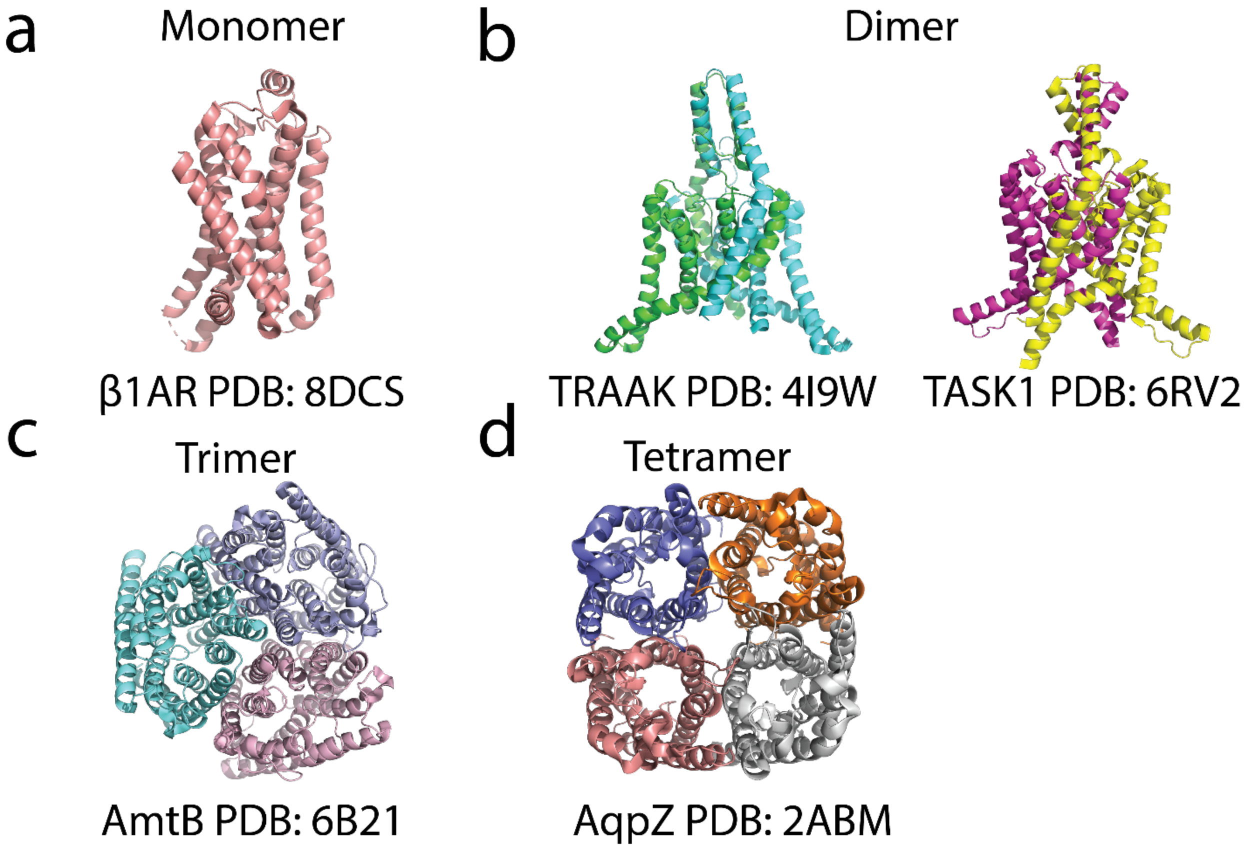

Supplementary Figure 20. Structures of membrane proteins studied in this work. a β1AR; **b** TRAAK and TASK1; **c** AmtB; **d** AqpZ

Supplementary Table 1. Detergent name, abbreviation, and their reported critical micelle concentration.

Abbreviation	IUPAC	CMC (mM)*	CMC (w/v%)
**C8E4**	Tetraethylene glycol monooctyl ether	~ 8 mM	0.25%
**C10E5**	Pentaethylene Glycol Monodecyl Ether	~ 0.81mM	0.03%
**DM**	n-Decyl-α-D-Maltopyranoside	~ 1.66 mM	0.08%
**DDM**	n-Dodecyl-β-D-Maltopyranoside	~ 0.17 mM	0.01%
**NG**	n-Nonyl β-D-glucopyranoside	~ 6 mM	~0.2%
**$(C4)_2$SPM**	N^1-(3-aminopropyl)-N^4-(3-(dibutylamino)propyl)butane-1,4-diamine	N/A	N/A
**C8SPM**	N^1-(3-aminopropyl)-N^4-(3-(octylamino)propyl)butane-1,4-diamine	N/A	N/A
**C10SPM**	N^1-(3-aminopropyl)-N^4-(3-(decylamino)propyl)butane-1,4-diamine	~ 13.67 mM	0.79%*
**C12SPM**	N^1-(3-aminopropyl)-N^4-(3-(dodecylamino)propyl)butane-1,4-diamine	~13.44 mM	0.81%*

* Except for C10SPM and C12SPM, CMC values were obtained from Anatrace.

Supplementary Table 2. Weighted average charge (Z_{avg}) extracted from UniDec for TRAAK with different additives.

Conc. (µM)	SPM	C8SPM	C10SPM	C12SPM	(C4)2SPM
apo	13.92 ± 0.16				
10	13.16 ± 0.97	13.92 ± 0.16	13.8 ± 0.2	13.62 ± 0.12	13.94 ± 0.19
100	13.17 ± 1.18	13.55 ± 0.21	13.26 ± 0.59	12.85 ± 0.29	13.62 ± 0.06
1000	12.33 ± 1.29	12.86 ± 0.88	11.19 ± 0.72	10.98 ± 0.79	13.3 ± 0.37
10000	9.82 ± 1.45	9.92 ± 0.1	7.82 ± 0.53	8.34 ± 0.63	10.83 ± 0.41

Supplementary Table 3. Weighted average charge (Z_{avg}) extracted from UniDec for AqpZ with different additives.

	SPM	C8SPM	C10SPM	C12SPM	(C4)2SPM
**apo**	14.9 ± 0.2				
**10**	14.87 ± 0.6	15.2 ± 0.3	14.93 ± 0.4	15.03 ± 0.48	14.91 ± 0.17
**100**	15.09 ± 0.46	14.99 ± 0.47	14.85 ± 0.41	15.17 ± 0.53	14.78 ± 0.16
**1000**	14.43 ± 0.35	14.67 ± 0.01	14.15 ± 0.55	14.07 ± 0.09	15.3 ± 0.71
**10000**	15.15 ± 0.22	11.12 ± 1.06	10.27 ± 0.18	10.21 ± 0.1	13.59 ± 1.08

Supplementary Table 4. Weighted average charge (Z_{avg}) mixed micelles containing C10SPM and either C10E5 or C8E4. A 100% denotes 1.6 mM for C10E5, 16 mM for C8E4 and 1.9 mM C10SPM.

%	TRAAK	AqpZ
**0**	11.7 ± 0.21	14.63 ± 0.00
**20**	9.79 ± 0.55	13.93 ± 0.70
**40**	9.06 ± 0.54	13.69 ± 0.94
**50**	8.47 ± 0.49	N/A
**60**	8.68 ± 0.46	12.57 ± 2.06
**80**	8.17 ± 0.26	11.80 ± 2.83
**100**	7.75 ± 0.53	9.20 ± 5.43

Supplementary Table 5. Instrument settings used for different samples on EMR Orbitrap mass spectrometer.

	DDM/DM		
	SID	HCD	Source DC
**AmtB**	70	100	50
**TASK1**	70	200	80
**TRAAK**	70	70	40
**AqpZ (DDM)**	60	60	50
**AqpZ (DM)**	40	60	50
**B1AR (DDM)**	50	60	50
**B1AR (DM)**	100	100	50

Supplementary Note 1:

Synthesis and characterization of SPM-derivatives.

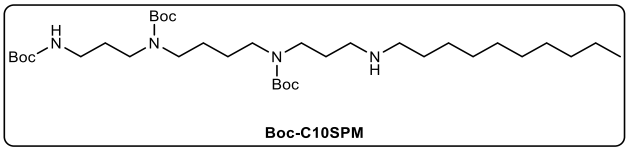

Boc-C10SPM. Compound **1** (0.899 g, 1.79 mmol, synthesized from spermine using a reported procedure[1]) was dissolved in a mixture of ethanol (25 mL) and acetic acid (1 mL) in a round-bottom flask. Decanal (0.233 g, 1.49 mmol) and sodium cyanoborohydride (0.141 g, 2.24 mmol) were added to the flask and the reaction mixture was stirred at room temperature for 24 h. 2M $HCl_{(aq.)}$ was added to the mixture until pH reached 2, followed by the addition of 1M $NaOH_{(aq.)}$ until pH reached 12. Ethanol was removed from the crude solution by rotary evaporator. The crude solution was then extracted with diethyl ether (3 × 50 mL). The organic layer was washed with water (3 × 100 mL), and brine (1 × 100 mL), before dried over Na_2SO_4. After removing the solvent on a rotary evaporator, the crude product was purified by column chromatography (SiO_2, CH_2Cl_2/MeOH/NH_4OH, 9/1/0.05) to afford Boc-C10SPM (0.891 g, 93%). ^{1}H NMR (400 MHz, $CDCl_3$, 298 K) δ = 3.30-3.07 (m, 10H), 2.83-2.59 (m, 2H), 2.05-1.85 (m, 4H), 1.72-1.58 (m, 4H), 1.54-1.37 (m, 33H), 1.36-1.11 (m, 14H), 0.87 (t, *J* = 6.7, 3H). ^{13}C NMR (126 MHz, $CDCl_3$, 298 K) δ = 156.06, 155.54, 79.58, 79.02, 53.39, 46.79, 43.84, 37.53, 31.91, 31.89, 31.86, 29.99, 29.69, 29.66, 29.63, 29.59, 29.57, 29.54, 29.48, 29.44, 29.34, 29.31, 29.26, 28.46, 28.44, 25.94, 22.67, 14.09. HRMS (+ESI): C35H70N4O6 [M+H]+ calcd. m/z = 643.5368, found m/z = 643.5357.

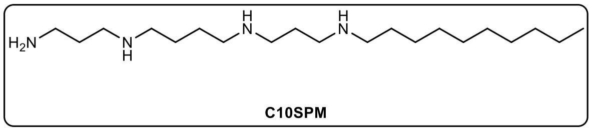

C10SPM. **Boc-C10SPM** (0.144 g, 0.224 mmol) was added to dry trifluoroacetic acid (5 mL) at room temperature and the mixture was stirred for 1 h. Excessive solvent was removed by purging nitrogen and the residual solution was thoroughly washed with diethyl ether to afford an off-white solid crude. Reverse-phase flash column chromatography [C18-SiO_2, gradient eluent: methanol/water = 0.05 to 0.5 with 2%(v/v) acetic acid] was performed to give **C10SPM** in its trifluoroacetate salt form as white solid (103 mg, 57%). ^{1}H NMR (500 MHz, D_2O, 298 K) δ = 3.23-3.10 (m, 14H), 2.18-2.13 (m, 4H), 1.85-1.82 (m, 4H), 1.75-1.72 (m, 4H), 1.44-1.32 (m, 14H), 0.94-0.91 (t, 3H). ^{13}C NMR (126 MHz, D_2O, 298 K) δ = 49.39, 48.52, 48.50, 46.04, 46.01, 45.75, 38.05, 32.67, 30.10, 29.94, 29.93, 29.64, 27.13, 26.95, 25.25, 24.26, 24.14, 23.54, 14.91. HRMS (+ESI): C24H46N4 [M+H]+ calcd. m/z = 343.3795, found m/z = 343.3790.

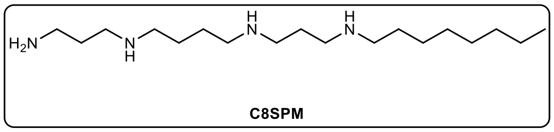

C8SPM. Compound **1** (0.096 g, 0.19 mmol) was dissolved in a mixture of ethanol (5 mL) and acetic acid (0.25 mL) in a round-bottom flask. Octanal (0.022 g, 0.17 mmol) and sodium cyanoborohydride (0.016 g, 0.26 mmol) were added to the flask and the reaction mixture was stirred at room temperature for 24 h. 2M $HCl_{(aq.)}$ was added to the mixture until pH reached 2, followed by the addition of 1M $NaOH_{(aq.)}$ until pH reached 12. Ethanol was removed from the crude solution by rotary evaporator. The crude solution was then extracted with diethyl ether (3 × 10 mL). The organic layer was washed with water (3 × 30 mL), and brine (1 × 50 mL), before dried over Na_2SO_4. After removing the solvent on a rotary evaporator, the crude product was transferred to a round-bottom flask and used directly for the deprotection step without further purification. To the round-bottom flask, dry trifluoroacetic acid (5 mL) was added at room temperature and the mixture was stirred for 2 h. Excessive solvent was removed by purging nitrogen and the residual solution was thoroughly washed with diethyl ether to afford an off-white solid crude. Reverse-phase flash column chromatography [C18-SiO_2, gradient eluent: methanol/water = 0.05 to 1 with 2%(v/v) acetic acid] was performed to give **C8SPM** in its trifluoroacetate salt form as white solid (65 mg, 44%). ^{1}H NMR (500 MHz, D_2O, 298 K) δ = 3.23-3.10 (m, 14H), 2.18-2.13 (m, 4H), 1.85-1.82 (m, 4H), 1.77-1.71 (m, 2H), 1.43-1.33 (m, 10H), 0.94-0.91 (t, 3H). ^{13}C NMR (126 MHz, D_2O, 298 K) δ = 49.38, 48.52, 48.50, 46.04, 46.01, 45.75, 38.05, 32.48, 29.63, 29.61, 27.12, 26.94, 25.27, 24.75, 24.27, 24.16, 23.46, 14.86. HRMS (+ESI): C18H42N4 [M+H]+ calcd. m/z = 315.3482, found m/z = 315.3478.

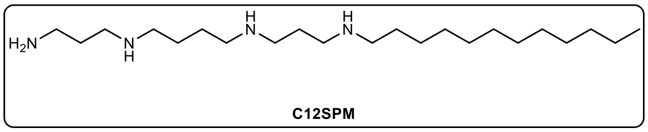

C12SPM. Compound **1** (0.141 g, 0.280 mmol) was dissolved in a mixture of ethanol (5 mL) and acetic acid (0.25 mL) in a round-bottom flask. Dodecanal (0.047 g, 0.25 mmol) and sodium cyanoborohydride (0.024 g, 0.38 mmol) were added to the flask and the reaction mixture was stirred at room temperature for 24 h. 2M $HCl_{(aq.)}$ was added to the mixture until pH reached 2, followed by the addition of 1M $NaOH_{(aq.)}$ until pH reached 12. Ethanol was removed from the crude solution by rotary evaporator. The crude solution was then extracted with diethyl ether (3 × 10 mL). The organic layer was washed with water (3 × 30 mL), and brine (1 × 50 mL), before dried over Na_2SO_4. After removing the solvent on a rotary evaporator, the crude product was transferred to a round-bottom flask and used directly for the deprotection step without further purification. To the round-bottom flask, dry trifluoroacetic acid (5 mL) was added at room temperature and the mixture was stirred for 2 h. Excessive solvent was removed by purging nitrogen and the residual solution was thoroughly washed with diethyl ether to afford an off-

white solid crude. Reverse-phase flash column chromatography [C18-SiO_2, gradient eluent: methanol/water = 0.05 to 1 with 2%(v/v) acetic acid] was performed to give **C12SPM** in its trifluoroacetate salt form as white solid (93 mg, 40%). ^1H NMR (500 MHz, D_2O, 298 K) δ = 3.23-3.10 (m, 14H), 2.26 (br, 3H), 2.20-2.12 (m, 4H), 1.85-1.82 (m, 4H), 1.77-1.70 (m, 2H), 1.44-1.34 (m, 18H), 0.94-0.91 (t, 3H). ^{13}C NMR (126 MHz, D_2O, 298 K) δ = 49.45, 48.57, 48.55, 46.09, 46.06, 45.80, 38.10, 32.76, 30.31, 30.29, 30.17, 30.06, 29.99, 29.70, 27.18, 27.01, 25.30, 24.30, 24.20, 23.61, 14.98. HRMS (+ESI): C22H50N4 [M+H]+ calcd. m/z = 371.4108, found m/z = 371.4103.

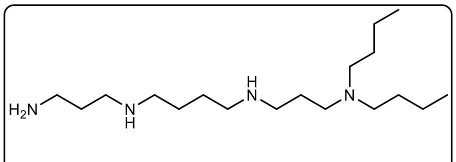

$(C4)_2$SPM

$(C4)_2$SPM. Compound **1** (0.27 g, 0.54 mmol) was dissolved in a mixture of ethanol (10 mL) and acetic acid (0.5 mL) in a round-bottom flask. Butanal (0.070 g, 0.98 mmol) and sodium cyanoborohydride (0.092 g, 1.47 mmol) were added to the flask in two portions with 1 h gap. The reaction mixture was stirred at room temperature for 24 h. 2M $HCl_{(aq.)}$ was added to the mixture until pH reached 2, followed by the addition of 1M $NaOH_{(aq.)}$ until pH reached 12. Ethanol was removed from the crude solution by rotary evaporator. The crude solution was then extracted with diethyl ether (3 × 15 mL). The organic layer was washed with water (3 × 50 mL), and brine (1 × 50 mL), before dried over Na_2SO_4. After removing the solvent on a rotary evaporator, the alkylated crude product was transferred to a round-bottom flask and used directly for the deprotection step without further purification. To the round-bottom flask, dry trifluoroacetic acid (5 mL) was added at room temperature and the mixture was stirred for 2 h. Excessive solvent was removed by purging nitrogen and the residual solution was thoroughly washed with diethyl ether to afford an off-white solid crude. Reverse-phase flash column chromatography [C18-SiO_2, gradient eluent: methanol/water = 0.05 to 1 with 2%(v/v) acetic acid] was performed to give **$(C4)_2$SPM** in its trifluoroacetate salt form as white solid (68 mg, 15%). ^1H NMR (500 MHz, D_2O, 298 K) δ = 3.32-3.14 (m, 4H), 2.19-2.13 (m, 16H), 1.85-1.82 (m, 4H), 1.76-1.70 (m, 4H), 1.48-1.40 (m, 4H), 1.01-0.98 (t, 3H). ^{13}C NMR (126 MHz, D_2O, 298 K) δ = 54.44, 51.07, 48.61, 48.53, 46.07, 45.99, 38.09, 26.70, 25.30, 24.31, 24.29, 22.14, 20.76, 14.28. HRMS (+ESI): C18H42N4 [M+H]+ calcd. m/z = 315.3482, found m/z = 315.3480.

Supplementary References

[1] M. R. Burns, S. A. Jenkins, M. R. Kimbrell, R. Balakrishna, T. B. Nguyen, B. G. Abbo, S. A. David, *Journal of Medicinal Chemistry* **2007**, *50*, 877-888.
